# Role of intrathecal chemotherapy in the management of meningeal carcinomatosis in patients with breast cancer

**DOI:** 10.31744/einstein_journal/2023AO0481

**Published:** 2023-12-12

**Authors:** Renata Tortato Meneguetti, Felipe José Silva Melo Cruz, Auro del Giglio

**Affiliations:** 1 Instituto Brasileiro de Controle do Câncer São Paulo SP Brazil Instituto Brasileiro de Controle do Câncer , São Paulo , SP , Brazil .; 2 Centro Universitário FMABC Santo André SP Brazil Centro Universitário FMABC , Santo André , SP , Brazil .

**Keywords:** Breast neoplasm, Meningeal carcinomatosis, Methotrexate, Injections, spinal, Palliative care, Metastases, Neoplasm, Survival, Neurologic manifestations

## Abstract

**Objective:**

To evaluate whether intrathecal chemotherapy improves clinical outcomes in patients with meningeal carcinomatosis.

**Methods:**

This retrospective cohort study included consecutive patients with breast cancer diagnosed with meningeal carcinomatosis. Clinical and treatment data were collected from the patients’ medical charts. The primary outcome was overall survival, and the secondary outcomes were time to neurological deterioration and reporting of clinical benefit. Logistic regression and Cox proportional hazard models adjusted for potential confounders were used to evaluate the clinical response and overall survival, respectively.

**Results:**

Overall, 109 female patients were included, 50 (45.9%) of whom received intrathecal chemotherapy with methotrexate and dexamethasone. The median treatment duration was 3 weeks (range, 1–13 weeks). Patients treated with intrathecal chemotherapy were more likely to report clinical benefit (74% *versus* 57.7%, adjusted odds ratio [OR] = 9.0, 95%CI=2.6–30.9, p<0.001). However, there was no difference in the time to neurologic deterioration (hazard ratio [HR] = 0.96, 95%CI= 0.57–1.59, p=0.86). Patients who received intrathecal chemotherapy did not show an increase in overall survival compared with that of patients who did not receive intrathecal chemotherapy (median overall survival = 1.8 months, 95%CI= 1.27–3.0 *versus* 2.5, 95%CI= 1.9–3.9, adjusted HR = 0.71, 95%CI= 0.41–1.22, p=0.21). There was a significant interaction between intrathecal chemotherapy and systemic treatment, and patients who received systemic therapy without intrathecal chemotherapy had better overall survival than that of the no-treatment group (adjusted HR = 0.38, 95%CI= 0.20–0.70, p=0.002).

**Conclusion:**

Intrathecal chemotherapy did not increase overall survival or time to neurological deterioration and should not preclude or postpone systemic treatments.

## Summary

In Brief

This retrospective cohort study included 109 patients. Statistical analyses were performed for the treatment Groups Intrathecal Chemotherapy versus No Intrathecal Chemotherapy and adjusted for potential confounders. The authors demonstrated that intrathecal chemotherapy did not increase the overall survival or time to neurological deterioration in patients with leptomeningeal metastatic breast cancer. The median survival time was less than 3 months in both groups.

## Abstract (key points)

Highlights

Intrathecal chemotherapy did not increase overall survival or time to neurologic deterioration.

Compared with no treatment, systemic therapy with no intrathecal chemotherapy afforded better overall survival.

Intrathecal chemotherapy should not postpone or preclude systemic treatment.

## Graphical abstract

**Figure:**
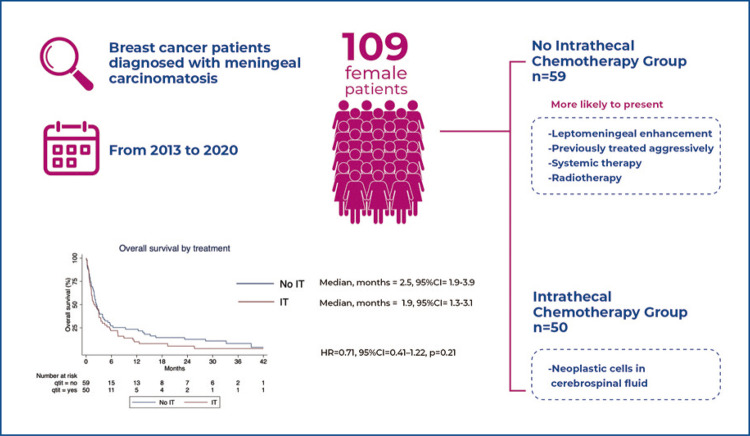

## INTRODUCTION

Meningeal carcinomatosis (MC) is defined as tumor cell infiltration into the meningeal layers and cerebrospinal fluid (CSF). ^( 1 )^ Breast cancer is the most common etiology of MC because of its high incidence. ^( 2 )^ Therefore, it is estimated that 2–5% of patients with breast cancer develop MC; however, this percentage may be underestimated owing to diagnostic challenges. ^( 3 )^ The clinical manifestations of MC are multifocal neurological signs and symptoms, including headache, vomiting, seizures, extremity weakness, paranesthesia, intestinal and urinary dysfunction, and diplopia. ^( 4 )^ The current recommendation for diagnosis is that every suspected case of MC should be investigated using neuroimaging examinations and CSF analysis. ^( 5 )^

In most cases, treatment is guided by expert opinions because of the lack of prospective and randomized clinical trials defining the best therapy. ^( 6 )^ Thus, treatment options include intrathecal chemotherapy, radiation therapy, systemic therapy, and palliative care, depending on the symptoms, disease volume, and functional status of the individual. ^( 7 )^

Regarding the survival of patients diagnosed with MC, the mean overall survival (OS) is estimated to be 2–4 months in treated cases. In untreated patients, neurological worsening evolved within 4–6 weeks. ^( 8 )^ Prognostic factors such as performance status, age, number of previous systemic treatments, tumor molecular profile, and biochemical characteristics of the CSF sample have been evaluated in previous studies. ^( 9 , 10 )^ However, there is no clear consensus on prognostic risk classification. ^( 11 )^

## OBJECTIVE

To evaluate the effect of intrathecal chemotherapy on the overall survival and clinical benefits in patients with metastatic breast cancer and meningeal carcinomatosis.

## METHODS

This was an observational retrospective cohort study of patients with breast cancer who were diagnosed with MC between 2013 and 2020. Clinical and treatment data were collected from the patients’ medical charts. The primary outcome was OS, defined as the time from MC diagnosis to death or loss to follow-up. Data were collected until January 2023, when surviving patients were administratively censored. The secondary outcomes were time to neurological progression and clinical benefits. Time to neurological progression was defined as the time from MC diagnosis to the deterioration of neurological symptoms, death, or loss to follow-up. Clinical benefit was defined as an improvement in symptoms reported by the patients (yes/no) during regular hospital visits.

Logistic regression and Cox proportional hazard models adjusted for potential confounders were used to evaluate clinical response and OS, respectively.

Adult patients (age: >18 years) diagnosed with MC by brain radiology examinations (computed tomography or magnetic resonance imaging) or the presence of neoplastic cells in the CSF were included. Patients with other solid or haematological malignancies were excluded.

The study was approved by the institutional ethical committee of *Instituto Brasileiro de Controle do Câncer* , CAAE: 32154920.8.0000.0072; # 4.069.647, where the study was conducted, and the need for written informed consent was waived.

### Statistical analysis

The clinical characteristics of the study population were presented by the treatment group. Summary statistics were constructed using frequencies and proportions for categorical data and means, medians, and ranges for continuous variables. Fisher’s exact test was used to analyze categorical variables, and an unpaired *t* -test was used for numerical variables.

Overall survival analysis used Kaplan-Meier estimates to determine the medians and 95% confidence intervals (95%CIs). The treatment groups were compared using hazard ratios (HRs) and the associated 95% CIs based on an unstratified Cox regression model. A clinical benefit analysis was performed using logistic regression. The dependent variable was whether the patient presented with symptom improvement as registered on the medical chart (dichotomous variable).

The Cox and logistic regression models were adjusted for potential confounders of the relationship between the outcome and the use of intrathecal chemotherapy by using propensity score. The following variables were included in the model: age (years), Eastern Cooperative Oncology Performance Status (ECOG, 0-1 or 2-4), molecular type (luminal, HER 2 positive, triple negative), presence of macroscopic central nervous system (CNS) metastasis, presence of visceral metastasis, concomitant treatment with radiotherapy, and systemic therapy). The Wald test was used to evaluate the presence of interactions between intrathecal chemotherapy and systemic treatment and between intrathecal chemotherapy and radiotherapy. Statistical significance was set at p<0.05 (2-tailed).

## RESULTS

Overall, 109 female patients were included, of whom 50 (45.9%) received intrathecal chemotherapy with methotrexate (12mg) and dexamethasone (4mg) twice weekly. Patients in the Intrathecal Chemotherapy Group were more likely to have neoplastic cells in the CSF (96%). The No Intrathecal Chemotherapy Group was more likely to have leptomeningeal contrast enhancement on brain images (81.4%), was more heavily treated (78.0%) of the patients received two or more lines of therapy), received more CNS radiation therapy (54.2%), and received more systemic treatment at the time of MC diagnosis (33.9%). Among patients who received intrathecal chemotherapy, five (10%) received concurrent systemic chemotherapy, and eight (16%) received concurrent radiotherapy. The full description of patient characteristics is summarized in table 1 .

**Table 1:** Clinical characteristics of the patients who did and did not receive intrathecal chemotherapy

	No Intrathecal Chemotherapy Group (n=59, 54.1%)	Intrathecal Chemotherapy Group (n=50, 45.9%)	p value
Age, years (mean, range)	56.6 (26–78)	54.4 (31–79)	0.33
Histology			
Ductal	48 (81.4)	40 (80)	
Lobular	6 (10.2)	8 (16)	0.50
Other	5 (8.4)	2 (4)	
Molecular type			
Luminal	49 (83)	35 (70)	
HER 2 positive	4 (6.8)	1 (2)	0.074
Triple negative	6 (10.2)	14 (28)	
Leptomeningeal contrast enhancement on MRI			
Absent	11 (18.6)	28 (56)	0.001
Present	48 (81.4)	22 (44)	
Neoplastic cells in CSF			
Absent	41 (69.5)	2 (4)	0.001
Present	18 (30.5)	48 (96)	
Metastasis			
Bone	54 (91.5)	32 (64)	0.001
Visceral	39 (66.1)	25 (50)	0.118
Macroscopic CNS	8 (13.5)	13 (26)	0.143
Up to two metastatic sites	29 (49.1)	27 (54)	0.701
ECOG PS			
0-1	37 (62.7)	30 (60)	0.84
2-4	22 (37.3)	20 (40)	
Previous line of chemotherapy			
Up to one line	13 (22.0)	21 (42.0)	0.037
Two or more lines	46 (78.0)	29 (58)	
Radiation therapy	32 (54.2)	8 (16)	0.001
Systemic treatment	20 (33.9)	7 (14)	0.025

Age presented as continuous variable (men, range). Other variables presented as categorical (frequency, proportion %).

CSF: cerebrospinal fluid; CNS: central nervous system; ECOG PS: Eastern Cooperative Oncology Performance Status Scale.

**Figure 1:**
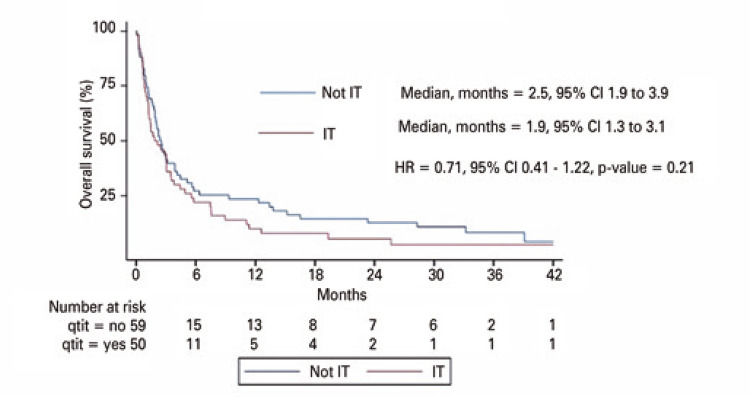
Overall survival by treatment

The median duration of treatment was three weeks (range: 1 – 13 weeks) and the median follow-up time was 2.3 months (range 0.1 – 54.1 months). Intrathecal chemotherapy did not increase OS compared with that in the No Intrathecal Chemotherapy Group (median OS = 1.9 months, 95%CI= 1.3–3.1 *versus* 2.5 months, 95%CI = 1.9–3.9, adjusted HR= 0.71, 95%CI= 0.41–1.22, p=0.21) ( Figure 1 ).

There was a significant interaction between intrathecal chemotherapy and systemic treatment, and patients who received systemic therapy without intrathecal chemotherapy had better overall survival than those who did not receive any treatment (adjusted HR= 0.38, 95%CI = 0.20–0.70, p=0.002). However, the benefit among those who received both intrathecal chemotherapy and systemic therapy was not significant compared with those who did not receive treatment ( Table 2 ). There was no significant interaction between radiotherapy and intrathecal chemotherapy with regard to OS (HR= 1.13, 95%CI= 0.57–2.23, p=0.73).

**Table 2:** Cox proportional hazard model of overall survival by use of intrathecal chemotherapy

	Univariate analysis			Multivariate analysis*		

	HR	95%CI	p value	HR	95%CI	p value
All patients	1.29	0.87–1.91	0.21	0.71	0.41–1.22	0.21
Patients treated with IT and systemic therapy	N/A		N/A	0.86	0.36–2.07	0.743
Patients not treated with IT and treated with systemic therapy	N/A		N/A	0.38	0.20–0.70	0.002

* Adjusted with propensity scores (variable included in the model: age, performance status, molecular type, central nervous system metastasis, visceral metastasis, previous lines of therapy, systemic therapy and radiotherapy and interaction term between intrathecal chemotherapy and systemic therapy). To calculate the hazard ratios, the reference category consisted of patients not treatment with systemic therapy or intrathecal chemotherapy.

HR: hazard ratios; 95%CI: 95% confidence intervals; IT: intrathecal chemotherapy.

**Figure 2:**
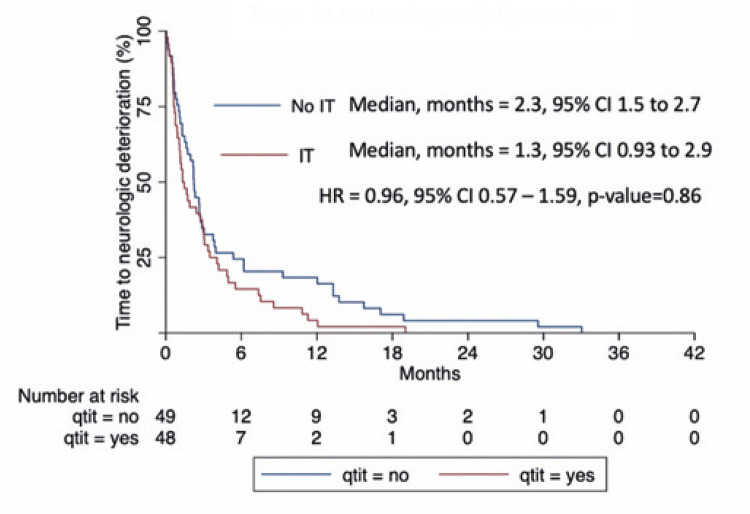
Time to neurologic deterioration

Patients treated with intrathecal chemotherapy were more likely to report clinical benefits (74.0%) *versus* 57.7%, adjusted OR= 9.0, 95%CI= 2.6–30.9, p<0.001) ( Table 3 ). However, there was no difference between the groups in terms of the time to neurologic deterioration: 1.3 months, 95%CI= 0.9–2.9 in the Intrathecal Chemotherapy Group, *versus* 2.2 months, 95%CI= 1.5–2.76, adjusted HR= 0.96, 95%CI= 0.57–1.59, p=0.86) ( Figure 2 and Table 3 ).

**Table 3:** Logistic regression analysis of the clinical benefit of intrathecal chemotherapy and Cox proportional hazard model for time to neurologic deterioration

				Univariate analysis			Multivariate analysis *		

		No Intrathecal Chemotherapy Group n (%)	Intrathecal Chemotherapy Group n (%)	OR	95%CI	p value	OR	95%CI	p value
Clinical benefit	No	25 (42.3%)	13 (26.0%)						
	Yes	34 (57.7%)	37 (74.0%)	2.1	0.9-4.7	0.076	9.0	2.6 – 30.9	p<0.001
				HR	95% CI	p value	HR	95% CI	p value
Time to neurologic deterioration	-	-	-	1.39	0.92 -2.10	p=0.113	0.96	0.57 – 1.59	p=0.86

*Adjusted with propensity scores (variables included in the model: age, performance status, molecular type, central nervous system metastasis, visceral metastasis, previous lines of therapy, systemic therapy, and radiotherapy).

95%CI: confidence interval; OR: odds ratio; HR: hazard ratios.

## DISCUSSION

The results of this study show that intrathecal chemotherapy does not increase overall survival or time to neurological deterioration compared to that in patients who were not treated with intrathecal chemotherapy. In both arms, the median survival time was less than 3 months, demonstrating the poor prognosis associated with MC in breast cancer.

The management of MC is challenging because there is no established optimal treatment. Several variables should be considered, including the patient’s performance status, presentation of the disease, previous therapy, treatment goals, and patient preferences. Azevedo et al described a Brazilian cohort of 60 MC breast cancer patients, with a median overall survival of 3.3 months from the time of diagnoses. Regarding prognostic factors related to survival, authors found that age, nuclear grade, hormonal and HER-2 status, CSF features, brain, lung, or bone metastasis, systemic or intrathecal chemotherapy, and radiotherapy had no impact in survival. However, in a multivariate analysis, histological grades 2 and 3 and poor PS were associated with poor outcomes. ^( 2 )^ Various treatment options such as systemic chemotherapy, intrathecal chemotherapy, chemotherapy, and radiation have been tested. ^( 12 )^ However, only a few randomized trials have been published on the treatment of patients with MC and the results are discordant.

Boogerd et al. evaluated the benefits of intraventricular chemotherapy in patients with breast cancer and MC. Systemic treatment and radiotherapy were administered to both arms. The trial included 35 patients, and results showed a median survival of 18.3 weeks associated with IT-CT and of 30.3 weeks associated with no IT-CT. The authors concluded that adding intraventricular chemotherapy did not lead to survival benefits or improved neurological responses and was associated with an increased risk of neurotoxicity. ^( 13 )^

Le Rhun et al. randomized 36 patients to receive systemic treatment or intrathecal chemotherapy in combination with systemic therapy. Focal radiotherapy was administered to six (16%) and three (8%) patients in the control and experimental groups, respectively. The median OS was 4.0 months (95%CI= 2.2–6.3) in the Control Group *versus* 7.3 months (95%CI= 3.9–9.6) in the experimental group (HR= 0.85, 95%CI= 0.53–1.36, p=0.51). Although there was a numerical difference in the median survival, the results were not statistically significant. One major limitation of this study was the imbalance between the arms, wherein patients in the control arm received more previous lines of treatment. ^( 14 )^

Patient selection remains a challenge in MC trials. In our study, we adjusted for imbalances, including the main confounders such as age, performance status, previous lines of therapy, concomitant radiation, and systemic treatment. However, owing to the retrospective nature of the study, the results may still be biased owing to unknown confounders.

All patients treated with intrathecal chemotherapy in our study received methotrexate and dexamethasone regardless of HER2 status. Targeted therapies, such as trastuzumab, showed improvement in the OS of patients with HER2-positive breast cancer. However, the actions of these therapies within the CNS remain questionable owing to their poor CNS penetrance. Even when the blood-brain barrier is disrupted owing to a local disease or following CNS radiation, trastuzumab cannot reach therapeutic concentrations in the CSF. ^( 15 )^ The administration of intrathecal trastuzumab has been investigated in several studies. A meta-analysis of 24 articles indicated that intrathecal chemotherapy with trastuzumab in patients having HER2-positive breast cancer with MC might be safe and effective; however, further prospective studies are needed. ^( 12 )^

Regarding systemic therapies, few clinical studies and case reports have evaluated MC, and their results are discordant. ^( 16 )^ Systemic therapy with drugs such as capecitabine, cyclophosphamide, 5-ﬂuorouracil, methotrexate, vinorelbine, and gemcitabine has been recommended for treating CNS metastasis. ^( 3 , 16 )^ A phase II study has evaluated the intracranial objective response rate in patients receiving abemaciclib. Although the study was negative for its primary endpoint, therapeutic concentrations of abemaciclib were achieved in brain metastatic tissue and CSF. ^( 17 )^

Sensitivity analysis of systemic treatment and intrathecal chemotherapy showed an effect modification of OS by using systemic therapy. Patients treated with systemic therapy without intrathecal chemotherapy had a 62% increase in survival compared to those who were not ( Table 2 ). In contrast, patients treated with both intrathecal chemotherapy and systemic therapy showed no benefit in terms of OS, suggesting that intrathecal chemotherapy could be deleterious. One limitation of this analysis is that we did not identify whether the patient had received chemotherapy or hormonal therapy.

Radiotherapy has been established to treat bulky diseases, symptomatic areas, and cauda equina syndrome. ^( 10 )^ It has the potential to provide rapid symptom relief and should be strongly considered, particularly in patients presenting with neurological deficits. ^( 18 )^ In our study, there was no effect modification of radiotherapy on OS by intrathecal chemotherapy.

The study has a few limitations. Despite the adjusted OR for improvement in clinical symptoms in the group of patients treated with intrathecal chemotherapy, this result has limited value in clinical practice because it was collected based on medical chart registration and the dichotomous nature of this endpoint (yes/no). A more appropriate endpoint for future studies should be a quality-of-life assessment based on patient-reported responses to questionnaires. Another limitation of our study is that we did not evaluate treatment toxicity, which is a vital issue, especially in patients with metastasis. Intrathecal chemotherapy can cause systemic side effects and discomfort owing to frequent lumbar punctures. ^( 13 )^ We also do not have data on whether there is a difference between the groups regarding the time from metastatic disease to the diagnosis of leptomeningeal metastasis, a potential biomarker of different patterns of disease progression.

Because of the heterogeneous and conflicting data, each patient diagnosed with breast cancer and MC requires a multidisciplinary and multimodal treatment approach with systemic therapy, radiotherapy, intrathecal therapy, and supportive care. ^( 13 )^ Our study showed that intrathecal chemotherapy did not improve relevant clinical outcomes in these patients.

## CONCLUSION

Intrathecal chemotherapy did not increase overall survival or time to neurological deterioration and should not preclude or postpone systemic treatments.
